# Leveraging STING, Batf3 Dendritic Cells, CXCR3 Ligands, and Other Components Related to Innate Immunity to Induce a “Hot” Tumor Microenvironment That Is Responsive to Immunotherapy

**DOI:** 10.3390/cancers14102458

**Published:** 2022-05-16

**Authors:** Robin Reschke, Daniel J. Olson

**Affiliations:** 1 Department of Pathology, University of Chicago, Chicago, IL 60637, USA; 2 Department of Medicine, University of Chicago, Comprehensive Cancer Center, Chicago, IL 60637, USA; daniel.olson2@uchospitals.edu

**Keywords:** chemokines, tumor microenvironment, dendritic cells

## Abstract

**Simple Summary:**

The conceptualization of a “hot” tumor microenvironment has been characterized by mainly T cell infiltration, whereas, “cold” or “immune-deserted” tumors were seen as lacking T cells. However, the presence of antigen-presenting myeloid cells is equally important. In particular, Batf3-lineage DCs are highly efficient in priming and recruiting effector T cells.

**Abstract:**

In a T-cell-inflamed phenotype, tumor eradication works best and is potentiated by immunotherapy such as checkpoint blockade. However, a majority of patients die despite receiving immunotherapy. One reason is insufficient T cell priming and infiltration in the tumor. Nature provides us with innate immune mechanisms in T-cell-inflamed tumors that we can adopt for more personalized immunotherapy strategies. Tumor sensing through innate signaling pathways and efficient antigen-presenting possess a significant role in bridging innate and adaptive immunity and generating a T-cell-inflamed tumor. One approach to strengthen these innate immune mechanisms is to deliver innate immune factors such as STING or activated DCs into the tumor microenvironment, in particular in patients resistant to checkpoint blockade. CD103^+^ DCs are integral for priming and recruiting of effector T cells. The presence of myeloid-cell-derived CXCL9 and CXCL10 in the tumor microenvironment can predict response to immunotherapy. We outline recent preclinical and clinical approaches to deliver these crucial components into the tumor microenvironment.

## 1. Introduction 

A T-cell-inflamed tumor is associated with better outcome to immunotherapy in metastatic cancer [1,2,3]. In order to create a T-cell-inflamed tumor microenvironment, a close interaction of the innate immune system with T cells is warranted. Of particular importance is tumor sensing through the STING (STimulator of INterferon Genes) pathway in antigen-presenting cells (APCs). Among APCs, dendritic cells are the most efficient cells in internalizing and presenting tumor antigens on MHC class I molecules to CD8^+^ T cells. In particular, Batf3-lienage DCs (basic leucine zipper transcription factor ATF-like 3 expressing dendritic cells) are required for successful cross-priming of effector T cells in the tumor draining lymph node [4,5]. DCs develop from bone-marrow-derived precursor cells and depend on the essential hematopoietic growth factor FMS-like tyrosine kinase 3 ligand (Flt3L). Tumor associated macrophages (TAMs) exhibiting an M1 phenotype are usually more abundant in the tumor microenvironment than DCs and can also present tumor antigens, though are generally less efficacious [6]. Myeloid cells are also involved in trafficking of tumor antigen specific T cells to the tumor microenvironment by releasing CXCR3-ligands. Whereas, Batf3 DCs can be recruited to the tumor bed through the chemokine (C-C motif) ligand 5 (CCL5) and XCL1 (also known as ATAC, lymphotactin, or SCM-1) produced by NK cells [7,8,9]. Sophisticated delivery systems for key innate immune factors such as bispecific antibodies, nanoparticles, fusion proteins, or viral vectors are on the edge of being translated into the clinic. Unwanted immune-related adverse events could be minimized by these targeted strategies. Ultimately, leveraging innate immunological pathways can lead to a more personalized immunotherapy. Future immunotherapy agents are likely to synergize with well-established antibodies for checkpoint blockade by enhancing infiltration with crucial immune cells such as activated Batf3 DCs and tumor-antigen-specific CD8+ T cells. 

## 2. How Are Endogenous T Cells Successfully Recruited into The Tumor Microenvironment?

**Figure 1:**
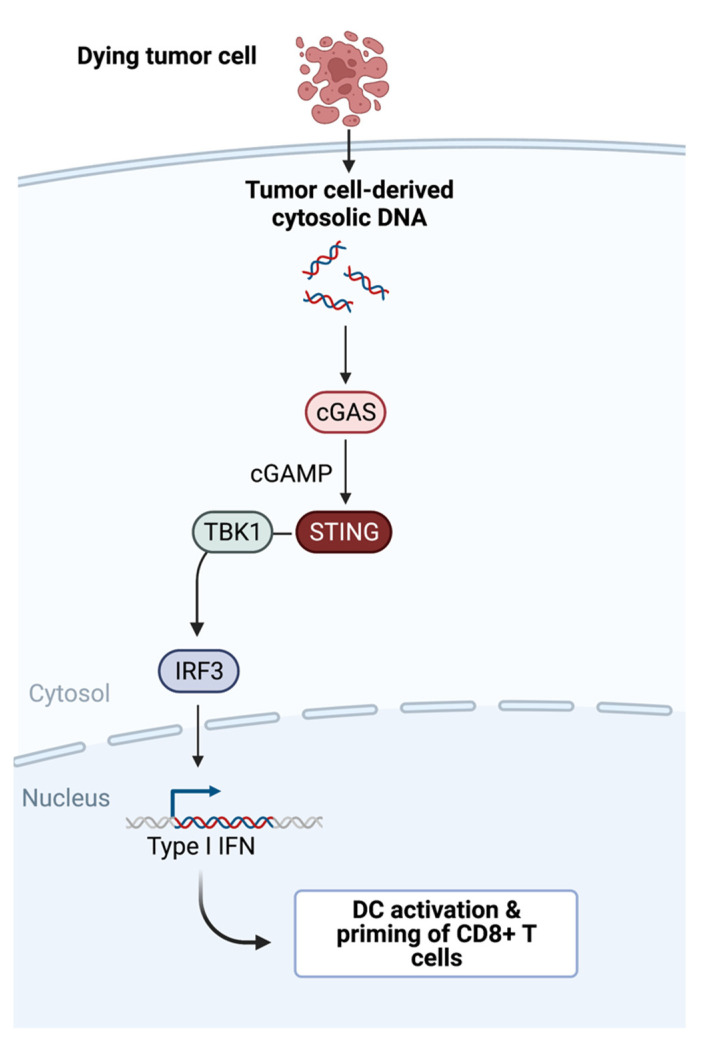
**STING pathway**: tumor-cell-derived DNA triggers the cGAS-cGAMP-STING signaling pathway and an innate immune response resulting in DC maturation and CD8+ T cell priming downstream of type I IFN production.

Non-T cell inflamed tumors have a set of immunogenic tumor antigens that are comparable to that of T cell inflamed tumors [10]. Thus, antigenicity is not the main reason for CTL infiltration. However, non-T cell inflamed tumors are lacking transcriptional markers for Batf3 DCs. Innate sensing of tumor derived factors is essential for the recognition of cancer by the immune system. One well-described signaling pathway is the STING pathway. Tumor infiltrating antigen presenting cells (APCs) can sense tumor-derived cytosolic DNA via cyclic-GMP-AMP synthase (cGAS) which activates the protein STING [5]. Activated STING results in the production of type I Interferons (IFNs) and maturation of Batf3 DCs downstream of the recruitment of TBK1 and the transcription factors IRF3 and NF-κB [11] (Figure 1). 

**Figure 2:**
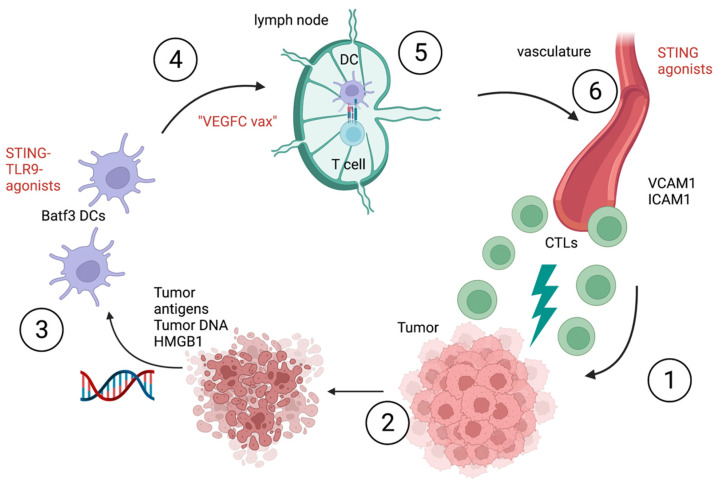
“Cancer–immunity cycle”. 1: CTL-mediated tumor killing; 2: tumor antigen release, release of HMGB1 and tumor-derived DNA; 3: tumor antigen uptake by Batf3 DCs, tumor-derived DNA and HMGB1 sensing via STING, TLR9 and TLR4, activation and maturation of Batf3 DCs; 4: migration of antigen-presenting Batf3 DCs through lymphatic vessels to the tumor-draining lymph node following a chemokine gradient of the CCR7-ligands CCL19 and CCL21; 5: DCs prime CD8+ T cells via a MHC-I–antigen complex, clonal expansion of tumor antigen specific T cells; 6: CTLs migrate via blood and exit the vessels into the tumor. Crawling along the endothelial wall and diapedesis are facilitated through the adhesion molecules VCAM1 and ICAM1. Possible interventions: STING−, TLR9− agonists and “VEGFC vax” enhancing cross-priming and vasculature normalization.

An alternate innate tumor sensing pathway involves the high-mobility-group box 1 (HMGB1) alarmin protein. This protein was secreted by dying tumor cells after radio- and chemotherapy and interacts with Toll-like receptor 4 (TLR4) and its adaptor MyD88 expressed by DCs [12,13]. The HMGB1 and TLR4 interaction improved antigen presentation by slowing down degradation of phagocytic cargo [12]. A subsequent report argues that TLR9 is involved sensing tumor-derived DNA, DC maturation, and CTL priming [13]. TLR9^−/−^ mice treated intratumorally with FITC-labeled E7 peptide and chemotherapy had significantly less FITC+ DCs in the tumor draining lymph nodes [14]. The CD11c+ DCs expressed less co-stimulatory molecules CD40 and CD80 compared to wild type (WT) mice. Furthermore, the frequency of CD8+ T cells was decreased in TLR9^−/−^ mice and they produced significantly less IFN-γ [14]. Matured CCR7 bearing CD103^+^ DCs take up cancer antigens and migrate through lymphatic vessels to the tumor draining lymph node following a chemokine gradient of CCL19 and CCL21 [15,16,17]. In the lymph node, CD103^+^ DCs cross-present internalized cancer antigens on MHC I molecules to naïve CD8+ T cells [18]. Tumor antigens are carried in small vesicles inside DCs that can also be transferred among DC subsets enabling also resident CD8α+ DCs to prime CD8+ T cells [19]. Recent results have shown that also CD11b+ conventional DCs can present tumor-derived antigens on MHC class I molecules upon interferon stimulation and thus contribute to anti-tumor CD8+ T cell immunity [20]. Activated CD8+ T cells expand clonally and then migrate through the blood stream to the tumor microenvironment. Integrin α4β1 (VLA-4) and integrins α4 and αLβ2 (LFA-1) on effector T cells bind to their endothelial ligands VCAM-1 and ICAM-1 which facilitates trans-endothelial migration and diapedesis [21]. Additionally, the interaction of LFA-1 and ICAM-1 contributes to the maturation of the cytotoxic immune synapse and effector function of CTLs [22]. CTLs can bind and kill their target cancer cells via specific T cell receptors which interact with the compatible antigen–MHC-I complex on the cancer cells [23]. Thus, new cancer antigens and DNA are released from the dead cancer cells and the “cancer–immunity cycle” begins anew. Within the “cancer–immunity cycle” there are multiple points of possible pharmacological interventions (Figure 2). 

### Clinical Applications

Clinical applications built on the foundation of this work include a phase 1b study, a combination of Pembrolizumab with vidutolimod, a TLR9-agonist, given intratumorally to 44 patients with resistance to prior anti-PD-1 treatment resulted in durable response in ¼ of the treated patients underlined by an increased IFNγ production [24]. In vivo and in vitro data have shown that STING agonists such as 5,6-dimethylxanthenone-4-acetic acid (DMXAA) or cyclic dinucleotide (CDN)-based STING agonists support DC maturation, facilitate CD8+ T cell cross-priming, and increase production of type I IFNs, TNF-α, and other innate cytokines [25]. However, DMXAA is not able to bind human STING [26]. Intratumoral administration of STING was favored in the past because of metabolic instability of CDN-based STING agonists [27]. Two recent reports propose alternate administration routes for the more stable STING agonists named MSA-2 and SR-717 configured in a closed confirmation [28,29] (Table 1). MSA-2 is administered orally and SR-717 systemically. Both agonists achieved cross-priming and tumor control in mice. Additionally, exosome delivery mechanisms for STING agonists are also in clinical development; this approach may help to overcome the direct cytotoxicity observed with initial clinical STING agonists [30]. Clinical trials for intravenous application of STING are ongoing (NCT04420884, NCT04096638, NCT04609579 and NCT04592484). Apart from DCs, tumor endothelial cells can also produce type I IFNs upon STING activation [31]. Endothelial STING expression correlated with CTL infiltration into the tumor in murine and human tissue and better overall survival [32]. In the mouse model, intratumorally administered STING upregulated genes for the vascular adhesion molecules Vcam and Icam, decreased hypoxia, reduced blood vessel density, and induced blood vessel maturation through increased pericyte coverage [32]. Other reports described tumor vasculature destruction through either high IFN-β-levels injected into the tumor or through STING agonists via TNF-α-production [25,33]. Overall, STING agonists in the right concentration can suppress angiogenesis/malformation and contribute to vascular normalization. Tumor antigen specific T cells need functional blood vessels within the tumor in order to migrate from the tumor draining lymph node to the tumor microenvironment. Conversely, antigen-loaded migratory DCs travel via lymph vessels from the tumor bed to the tumor draining lymph nodes. B16 melanoma bearing mice with blocked vascular endothelial growth factor receptor 3 (VEGFR3) signaling and a lack of lymphatic vessels showed significantly less trafficking of DCs to the draining lymph nodes, reduced immune cell infiltrates, and inflammatory cytokines in the tumor microenvironment [34]. Inducing lymph angiogenesis with a vaccine overexpressing VEGF-C (“VEGFC vax”) resulted in increased infiltration of CD8α+ cross-presenting DCs and CD8+ T cells and hence better tumor control in combination with anti-PD-1 in a B16 melanoma mouse model [35]. 

## 3. Batf3 DCs Initiating a Chemokine-Cytokine Network in the TME

Another important mechanism for immune cell infiltration into the tumor microenvironment is chemoattraction through chemokines. CXCL9, CXCL10, and CXCL11 (in humans) bind to the CXCR3-receptor on T cells. CXCL9 and CXCL10 enhanced migration of T cells in vivo and in vitro and were associated with T-cell-inflamed tumors in a gene expression analysis [2]. Type I IFN induces the expression of CXCL9 and CXCL10 [36]. Preclinically, Batf3 DCs were characterized as a predominant source for CXCL9 and CXCL10 [4]. The presence of Batf3 DCs, CXCL9 and CXCL10 in the tumor microenvironment independently correlated with T cell infiltration [4]. In melanoma patients, baseline levels of CXCL9, CXCL10, and CXCL11 were associated with response to anti-PD-1 therapy [37]. According to single-cell RNA sequencing data from patients with melanoma, head and neck, and lung cancers who underwent checkpoint blockade therapy, these chemokines were primarily produced by macrophages [37]. DCs such as CD103^+^ DCs were the second strongest cellular source for CXCL9/10/11 among the measured immune cells. In vitro, melanoma cell lines M537 produced CXCL9 and CXCL10 which was associated CD8+ effector T cells [2]. In a recently published work with in situ staining of RNA transcripts and proteins in baseline biopsies of melanoma patients, we found that the major cellular source of CXCL10 was indeed CD45+ immune cells in humans [38]. However, cancer cells were also able to produce CXCL10 to a lesser extent in some patients before commencing therapy with anti-PD-1. The baseline CXCL10 production of CD45+ immune cells and Sox10+ both correlated significantly with response to immunotherapy [38]. 

**Figure 3:**
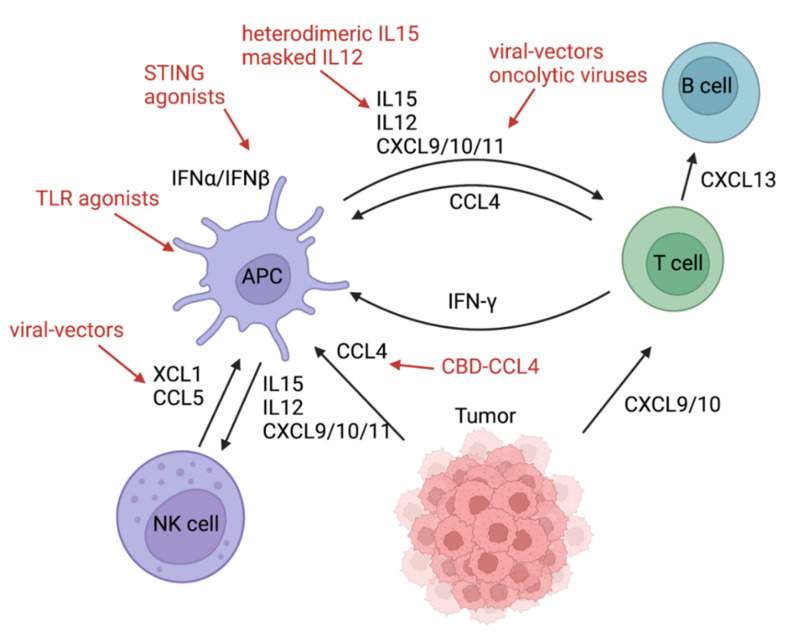
Chemokine/cytokine network in the tumor microenvironment. Tumor-derived CCL4 can attract Batf3 DCs, and CXCL9/10 can recruit cytotoxic T cells to the tumor microenvironment. Natural Killer Cells attract Batf3 DCs via XCL1 and CCL5. CCL4 can be administered with a fusion protein consisting of CCL4 and the collagen-binding domain (CBD) of von Willebrand factor and XCL1 with a viral vector. APCs (in particular Batf3 DCs) can produce CXCL9/10/11, IL12, and IL15 and can recruit and activate NK and cytotoxic T cells. In turn, IFN-γ produced by CTLs can stimulate APCs. APCs are activated by STING or TLR agonists, which can contribute to CXCL9/10/11 production via IFNα/β. CXCL9/10/11 can be induced by oncolytic viruses or delivered with the help of virus-based vectors.

In vitro, cancer cells also produced CCL4, which led to the migration of CD103^+^ DCs [8]. The corresponding receptor on DCs is CCR5 [39]. Tumor-derived CCL4 can recruit CD103^+^ into the tumor microenvironment. Conversely, activated B-catenin signaling and ATF3 transcription suppressed CCL4 transcription [8]. CCL4 expression levels were increased significantly in T-cell-infiltrated tumors [2], arguing for successful cross-priming and recruitment of T cells by Batf3 DCs. In a murine model, XCL1 and CCL5 were mainly produced by NK cells and attracted conventional type 1 dendritic cells (cDC1) into the tumor microenvironment [7] (Figure 3). XCL1 binds to its chemokine receptor XCR1 which is expressed on Batf3 DCs [40,41]. In humans, the gene signature of XCL1 was associated with better survival and correlated with a NK cell and a cDC1 signature [7]. NK cells themselves can also be recruited via CXCL9-11 because they express CXCR3 on their surface [42]. In the TME, they are involved in killing cancer stem cells and thereby prevent early and late metastases [43,44]. NK cells additionally secrete IFNγ and TNFα [42]. For optimal tumor control, it has been found that IL12 exerted from Batf3 DCs is the premise for effective NK cell function and production of IFNγ [45]. Only Batf3 DCs were able to produce sufficient IL12 and achieve NK cell-mediated control of metastasis. Next to IL12, IL15 represents another important cytokine that can be produced by dendritic cells upon activation with type I IFN [46]. IL15 in turn activates NK cells and cytotoxic T cells and increases the production of IFNγ. Additionally, it boosts the production of CXCL9-11 in Batf3 DCs, most likely indirectly through increased IFNγ production [47]. Interestingly, a very recent study found that “exhausted” T cells in the tumor microenvironment that were actively recruited via CXCL9 and CXCL10 can also be considered chemokine-producing cells. They express checkpoint molecules such as LAG3 but also secrete CCL4 and CXCL13 and thereby attract more Batf3 DCs and B cells into the tumor microenvironment [48,49]. Accumulated B cells can form tertiary lymphoid structures and contribute to anti-tumor immunity. This complex chemokine/cytokine network starting with Batf3 DCs could potentially be targeted at various points with personalized medicine approaches, many of which are already being tested in clinical studies.

### 3.1. Inducing Chemokines and Cytokines

Injecting a virus-based vector expressing XCL1 and soluble Flt3L intratumorally improved tumor control in MC38- and B16-bearing mice through the accumulation of cross-presenting Batf3 DCs in the tumor draining lymph node [50]. Synergistic effects with anti-PD-1, anti-CD137, or anti-CTLA-4 mAbs were described. Interestingly, Batf3 knockout mice were missing these anti-tumoral effects. Another group exploited a different strategy to deliver the DC-chemoattractant CCL4 into the tumor microenvironment. Instead of injecting a viral-vector into the tumor directly, a fusion protein consisting of CCL4 and the collagen-binding domain (CBD) of von Willebrand factor were administered intravenously [51]. Increased infiltration with CD103^+^ DCs and CD8+ T cells was noted in the tumor microenvironment. By binding the collagen of the tumor stroma specifically, off-target side effects were not observed in this B16 melanoma mouse model. Combination therapy of CBD-CCL4 and checkpoint blockade improved survival significantly [51]. Interestingly, blocking only CXCR3 led to a loss of tumor control through this combination therapy, underpinning the importance of Batf3 DCs and CXCR3 ligands for the recruitment of CTLs and tumor killing. The CXCR3 ligands CXCL9 and CXCL10 also have been successfully delivered via viral-vectors in colon and melanoma mouse models [52,53]. The intra-tumoral delivery resulted in increased CTL infiltration and better tumor control. CXCL9 delivery with a viral-vector was combined with the immunostimulatory factor OX40 ligand (OX40L)/tumor necrosis factor superfamily member 4 (TNFSF4) and anti-PD-1 therapy [53]. Furthermore, oncolytic viruses can function as strong inducers of CXCL9 and 10 in the tumor microenvironment [54,55]. One group designed an oncolytic virus overexpressing CXCL11 and proved increased frequency of tumor infiltrating lymphocytes in a murine tumor model [56]. The viral enhancement of the CXCR3 ligands expression worked synergistically with checkpoint blockade in all animal models [54,55,56]. Clinical trials are starting to leverage these concepts to treat human cancers. A current trial makes use of an oncolytic virus which actively expresses an FAP-TAc antibody, CXCL9, CXCL10, and IFNα (NCT04053283). CXCL10 can be indirectly upregulated by endogenous DNA sensing pathways such as STING. This has been shown inversely by blocking IFNAR1 which downregulated CXCL10 expression in vitro in a colon cancer model [57]. In vitro bone-marrow-derived DCs expressed significantly more CXCL9 upon STING activation [5]. CXCL9/10 expression downstream of STING activation is mediated in a IFNα/IFN-β-dependent fashion. Thus, in vitro experiments have shown that IFN-β treatment induced CXCL10 expression of melanoma cells [58] and DCs stimulated with IFNα/IFN-β produced significant amounts of CXCL9 and CXCL10 [41]. Blockade with an anti-PD-1 antibody enhanced proliferation of T cells and thereby increased expression of IFN-γ in the tumor microenvironment which in turn increased production of CXCL10 (Interferon gamma-induced protein 10) [59]. A combination treatment consisting of chemotherapy (cisplatin), COX-2 inhibitor (celecoxib), type I IFN, and a TLR3 agonist (rintalomid) also led to successful induction of CXCL9-11 in patients with epithelial ovarian cancer [60]. In the future, this approach will be combined with DC vaccination to achieve optimal tumor control of “cold” tumors via cytotoxic T cell recruitment. In mice, the administration of heterodimeric IL-15 resulted in increased levels of XCL1, IFNγ, and CD103^+^ DCs, as well as CXCL9/10 production, and the recruitment of NK and T cells [47]. Another attractive avenue would be to leverage radiation therapy to induce cytokine/chemokine production. Radiation therapy in melanoma-bearing mice induced Type I IFN and CXCL10 production by myeloid cells because cytosolic dsDNA released from dying cancer cells activates the STING pathway in dendritic cells [61].

All these approaches constitute important new treatment avenues for “cold” tumors. However, these strategies of chemokine and cytokine induction only work well when key innate immune cells such as Batf3 DCs are present in the TME. Another limiting factor is the right formulation and delivery of these components. Systemic application or an increased dosage of CXCL9-11, for example, may lead to unwanted immune-related adverse events. Severe adverse events can lead to the discontinuation of checkpoint blockade therapy. Innovative delivery strategies might help circumvent toxicity. In a mouse model, masking IL12 by fusing it to a domain of the IL12 receptor prevented systemic toxicity despite intravenous application [62]. With this modification, the anti-tumor effect remained intact.

### 3.2. Adverse Effects Caused by Chemokines and Cytokines

Systemic upregulation of CXCL9/10/11 can contribute to inflammation and autoimmunity such as Alopecia areata, Vitiligo, autoimmune arthritis, type 1 diabetes, or adult-onset Still’s disease as well as immunotherapy-induced toxicity [62,63,64,65,66]. Patients with checkpoint inhibitor colitis showed an upregulation of CXCL9 and CXCL10 in the myeloid compartment measured with single-cell RNA sequencing [67]. Increased levels of CXCL9-11 in the blood during checkpoint blockade therapy were also associated with occurrence of irAE [68]. In vitro migration studies underlined that low concentrations of CXCL9 and CXCL10 produced by tumor endothelial cells promoted chemotaxis. However, high concentrations of CXCL9 and 10 produced by endothelial cells induced trans-endothelial migration (chemo-repulsion) of melanoma cells [69], arguing for a dose-dependent effect of the chemokine gradients. Therefore, targeted chemokine delivery has to be performed with caution. Nonetheless, it has potential to expand efficacy of checkpoint blockade when administered in the correct time, dosage, and directly into the tumor microenvironment. Similarly, systemic administration of cytokines can lead to severe and life-threatening toxicity. IL12, for example, results in an extensive production of systemic IFN-γ from NK cells [62]. Accordingly, potential off-tumor effects of systemic administration of cytokines must always be considered in clinical trial designs of these therapies.

**Table 1:** Induction and delivery of components related to the innate immune system that synergize with immunotherapy: summary of recent preclinical and clinical studies.

Components	Function	Species	Delivery Route/Therapeutic Agent	Year/Citation
**STING**	Sensing tumor-derived DNA, DC maturation, and CTL priming	mouse	systemic administration of SR-717 in a “closed” conformation	2020 [28]
		mouse	oral administration of MSA-2 in a “closed” conformation	2020 [29]
		mouse	engineered extracellular vesicle exogenously loaded with cyclic dinucleotide	2021 [30]
		human	intravenous infusion of TAK-676	ongoing NCT04420884
		human	intravenous infusion of SB 11285	ongoing NCT04096638
		human	intravenous infusion of SNX281	ongoing NCT04609579
		human	intratumoral injection of CDK-002	ongoing NCT04592484
**TLR9**	sensing tumor-derived DNA, DC maturation, and CTL priming	human	intratumoral injection of Vidutolimod	2021 [24]
		mouse	intratumoral injection CpG oligodeoxynucleotide (TLR9 ligand) and an antibody against OX40	2022 [70]
**TLR3**	maturation and CTL priming	human	intraperitoneal injection of rintalomid (TLR3 agonist), celecoxib, and cisplatin	2022 [60]
**VEGF**	local lymphangiogenesis, immune cell trafficking, and CTL activation	mouse	injection of “VEGFC vax”	2021 [35]
**XCL1+** **Flt3L**	DC recruitment and expansion	mouse	intratumoral injection of XCL1 and SFlt3L encoded in recombinant Semliki Forest virus-derived vectors	2018 [50]
**CCL4**	DC recruitment	mouse	intravenous administration of a fusion protein of CCL4 and the collagen-binding domain of von Willebrand factor	2019 [51]
**CXCL9/10/11**	CTL recruitment	mouse	CXCL9 and OX40L	2020 [53]
		mouse	intravenous injection of oncolytic vesicular stomatitis virus encodes CXCL9	2020 [55]
		mouse	genetically engineered mesenchymal stem cells producing CXCL10	2018 [71]
		humanized mouse	injection of CXCL10-producing SynNotch T cells	2021 [72]
		mouse	intravenous delivery of CXCL9/10/11 plasmids by nanoparticles	2022 [73]
		human	NG-641 is an oncolytic adenoviral vector which expresses a FAP-TAc antibody together with an immune enhancer module (CXCL9/CXCL10/IFNα).	ongoing NCT04053283
**IL12**	DC activation/IFNγ production of T and NK cells	mouse	intravenous injection of IL12 fused to a domain of the IL12 receptor	2022 [61]
**IL15**	DC recruitment/activation and downstream recruitment of CTLs/NK cells	mouse	intraperitoneal injection of heterodimeric IL-15	2020 [47]

## 4. Conclusions

Learning from the innate immune system and functional endogenous T cell priming, activation and recruitment in the tumor microenvironment can offer various leverage points to improve immunotherapy. Attempts to bring innate immune DNA sensing agonists, DC- and T-cell-chemoattracting factors or co-stimulatory molecules into the clinic are ongoing. Challenges lie in finding the right formulation, doses, delivery route, and combination for efficient agonists without causing systemic adverse events. Immune related adverse events could potentially be overcome by innovatively engineered delivery-systems such as nanoparticles or engineered oncolytic viruses. All the aforementioned strategies facilitate optimal activation and recruitment of CTLs into the tumor environment and, therefore, work well in conjunction with existing immunotherapies such as checkpoint blockade or adoptive cell therapy. Checkpoint blockade therapy is already used for many metastasized solid tumors but is also well accepted in the adjuvant setting for, e.g., melanoma patients [74].

## References

[1] Fridman W.H., Pagès F., Sautès-Fridman C., Galon J. (2012). The Immune Contexture in Human Tumours: Impact on Clinical Outcome. Nat. Rev. Cancer.

[2] Harlin H., Meng Y., Peterson A.C., Zha Y., Tretiakova M., Slingluff C., McKee M., Gajewski T.F. (2009). Chemokine Expression in Melanoma Metastases Associated with CD8 ^+^ T-Cell Recruitment. Cancer Res..

[3] Tumeh P.C., Harview C.L., Yearley J.H., Shintaku I.P., Taylor E.J.M., Robert L., Chmielowski B., Spasic M., Henry G., Ciobanu V. (2014). PD-1 Blockade Induces Responses by Inhibiting Adaptive Immune Resistance. Nature.

[4] Spranger S., Dai D., Horton B., Gajewski T.F. (2017). Tumor-Residing Batf3 Dendritic Cells Are Required for Effector T Cell Trafficking and Adoptive T Cell Therapy. Cancer Cell.

[5] Woo S.-R., Fuertes M.B., Corrales L., Spranger S., Furdyna M.J., Leung M.Y.K., Duggan R., Wang Y., Barber G.N., Fitzgerald K.A. (2014). STING-Dependent Cytosolic DNA Sensing Mediates Innate Immune Recognition of Immunogenic Tumors. Immunity.

[6] Liu J., Geng X., Hou J., Wu G. (2021). New Insights into M1/M2 Macrophages: Key Modulators in Cancer Progression. Cancer Cell Int..

[7] Böttcher J.P., Bonavita E., Chakravarty P., Blees H., Cabeza-Cabrerizo M., Sammicheli S., Rogers N.C., Sahai E., Zelenay S., Reis e Sousa C. (2018). NK Cells Stimulate Recruitment of CDC1 into the Tumor Microenvironment Promoting Cancer Immune Control. Cell.

[8] Spranger S., Bao R., Gajewski T.F. (2015). Melanoma-Intrinsic β-Catenin Signalling Prevents Anti-Tumour Immunity. Nature.

[9] Guilliams M., Ginhoux F., Jakubzick C., Naik S.H., Onai N., Schraml B.U., Segura E., Tussiwand R., Yona S. (2014). Dendritic Cells, Monocytes and Macrophages: A Unified Nomenclature Based on Ontogeny. Nat. Rev. Immunol..

[10] Spranger S., Luke J.J., Bao R., Zha Y., Hernandez K.M., Li Y., Gajewski A.P., Andrade J., Gajewski T.F. (2016). Density of Immunogenic Antigens Does Not Explain the Presence or Absence of the T-Cell–Inflamed Tumor Microenvironment in Melanoma. Proc. Natl. Acad. Sci. USA.

[11] Flood B.A., Higgs E.F., Li S., Luke J.J., Gajewski T.F. (2019). STING Pathway Agonism as a Cancer Therapeutic. Immunol. Rev..

[12] Apetoh L., Ghiringhelli F., Tesniere A., Obeid M., Ortiz C., Criollo A., Mignot G., Maiuri M.C., Ullrich E., Saulnier P. (2007). Toll-like Receptor 4–Dependent Contribution of the Immune System to Anticancer Chemotherapy and Radiotherapy. Nat. Med..

[13] Kang T.H., Mao C.-P., Lee S.Y., Chen A., Lee J.-H., Kim T.W., Alvarez R.D., Roden R.B.S., Pardoll D., Hung C.-F. (2013). Chemotherapy Acts as an Adjuvant to Convert the Tumor Microenvironment into a Highly Permissive State for Vaccination-Induced Antitumor Immunity. Cancer Res..

[14] Kang T.H., Mao C.-P., Kim Y.S., Kim T.W., Yang A., Lam B., Tseng S.-H., Farmer E., Park Y.-M., Hung C.-F. (2019). TLR9 Acts as a Sensor for Tumor-Released DNA to Modulate Anti-Tumor Immunity after Chemotherapy. J. Immunother. Cancer.

[15] Loef E.J., Sheppard H.M., Birch N.P., Dunbar P.R. (2021). Live-Cell Microscopy Reveals That Human T Cells Primarily Respond Chemokinetically Within a CCL19 Gradient That Induces Chemotaxis in Dendritic Cells. Front. Immunol..

[16] Martín-Fontecha A., Sebastiani S., Höpken U.E., Uguccioni M., Lipp M., Lanzavecchia A., Sallusto F. (2003). Regulation of Dendritic Cell Migration to the Draining Lymph Node: Impact on T Lymphocyte Traffic and Priming. J. Exp. Med..

[17] Roberts E.W., Broz M.L., Binnewies M., Headley M.B., Nelson A.E., Wolf D.M., Kaisho T., Bogunovic D., Bhardwaj N., Krummel M.F. (2016). Critical Role for CD103(+)/CD141(+) Dendritic Cells Bearing CCR7 for Tumor Antigen Trafficking and Priming of T Cell Immunity in Melanoma. Cancer Cell.

[18] Corrales L., Matson V., Flood B., Spranger S., Gajewski T.F. (2017). Innate Immune Signaling and Regulation in Cancer Immunotherapy. Cell Res..

[19] Ruhland M.K., Roberts E.W., Cai E., Mujal A.M., Marchuk K., Beppler C., Nam D., Serwas N.K., Binnewies M., Krummel M.F. (2020). Visualizing Synaptic Transfer of Tumor Antigens among Dendritic Cells. Cancer Cell.

[20] Duong E., Fessenden T.B., Lutz E., Dinter T., Yim L., Blatt S., Bhutkar A., Wittrup K.D., Spranger S. (2022). Type I Interferon Activates MHC Class I-Dressed CD11b+ Conventional Dendritic Cells to Promote Protective Anti-Tumor CD8+ T Cell Immunity. Immunity.

[21] Shulman Z., Cohen S.J., Roediger B., Kalchenko V., Jain R., Grabovsky V., Klein E., Shinder V., Stoler-Barak L., Feigelson S.W. (2012). Transendothelial Migration of Lymphocytes Mediated by Intraendothelial Vesicle Stores Rather than by Extracellular Chemokine Depots. Nat. Immunol..

[22] Franciszkiewicz K., Le Floc’h A., Boutet M., Vergnon I., Schmitt A., Mami-Chouaib F. (2013). CD103 or LFA-1 Engagement at the Immune Synapse between Cytotoxic T Cells and Tumor Cells Promotes Maturation and Regulates T-Cell Effector Functions. Cancer Res..

[23] Chen D.S., Mellman I. (2013). Oncology Meets Immunology: The Cancer-Immunity Cycle. Immunity.

[24] Ribas A., Medina T., Kirkwood J.M., Zakharia Y., Gonzalez R., Davar D., Chmielowski B., Campbell K.M., Bao R., Kelley H. (2021). Overcoming PD-1 Blockade Resistance With CpG-A Toll-Like Receptor 9 Agonist Vidutolimod in Patients With Metastatic Melanoma. Cancer Discov..

[25] Corrales L., Glickman L.H., McWhirter S.M., Kanne D.B., Sivick K.E., Katibah G.E., Woo S.-R., Lemmens E., Banda T., Leong J.J. (2015). Direct Activation of STING in the Tumor Microenvironment Leads to Potent and Systemic Tumor Regression and Immunity. Cell Rep..

[26] Conlon J., Burdette D.L., Sharma S., Bhat N., Thompson M., Jiang Z., Rathinam V.A.K., Monks B., Jin T., Xiao T.S. (2013). Mouse, but Not Human STING, Binds and Signals in Response to the Vascular Disrupting Agent 5,6-Dimethylxanthenone-4-Acetic Acid. J. Immunol..

[27] Gajewski T.F., Higgs E.F. (2020). Immunotherapy with a Sting. Science.

[28] Chin E.N., Yu C., Vartabedian V.F., Jia Y., Kumar M., Gamo A.M., Vernier W., Ali S.H., Kissai M., Lazar D.C. (2020). Antitumor Activity of a Systemic STING-Activating Non-Nucleotide CGAMP Mimetic. Science.

[29] Pan B.-S., Perera S.A., Piesvaux J.A., Presland J.P., Schroeder G.K., Cumming J.N., Trotter B.W., Altman M.D., Buevich A.V., Cash B. (2020). An Orally Available Non-Nucleotide STING Agonist with Antitumor Activity. Science.

[30] Jang S.C., Economides K.D., Moniz R.J., Sia C.L., Lewis N., McCoy C., Zi T., Zhang K., Harrison R.A., Lim J. (2021). ExoSTING, an Extracellular Vesicle Loaded with STING Agonists, Promotes Tumor Immune Surveillance. Commun. Biol..

[31] Demaria O., De Gassart A., Coso S., Gestermann N., Di Domizio J., Flatz L., Gaide O., Michielin O., Hwu P., Petrova T.V. (2015). STING Activation of Tumor Endothelial Cells Initiates Spontaneous and Therapeutic Antitumor Immunity. Proc. Natl. Acad. Sci. USA.

[32] Yang H., Lee W.S., Kong S.J., Kim C.G., Kim J.H., Chang S.K., Kim S., Kim G., Chon H.J., Kim C. (2019). STING Activation Reprograms Tumor Vasculatures and Synergizes with VEGFR2 Blockade. J. Clin. Investig..

[33] Spaapen R.M., Leung M.Y.K., Fuertes M.B., Kline J.P., Zhang L., Zheng Y., Fu Y.-X., Luo X., Cohen K.S., Gajewski T.F. (2014). Therapeutic Activity of High-Dose Intratumoral IFN-β Requires Direct Effect on the Tumor Vasculature. J. Immunol..

[34] Lund A.W., Wagner M., Fankhauser M., Steinskog E.S., Broggi M.A., Spranger S., Gajewski T.F., Alitalo K., Eikesdal H.P., Wiig H. (2016). Lymphatic Vessels Regulate Immune Microenvironments in Human and Murine Melanoma. J. Clin. Investig..

[35] Sasso M.S., Mitrousis N., Wang Y., Briquez P.S., Hauert S., Ishihara J., Hubbell J.A., Swartz M.A. (2021). Lymphangiogenesis-Inducing Vaccines Elicit Potent and Long-Lasting T Cell Immunity against Melanomas. Sci. Adv..

[36] Fuertes M.B., Woo S.-R., Burnett B., Fu Y.-X., Gajewski T.F. (2013). Type I Interferon Response and Innate Immune Sensing of Cancer. Trends Immunol..

[37] House I.G., Savas P., Lai J., Chen A.X.Y., Oliver A.J., Teo Z.L., Todd K.L., Henderson M.A., Giuffrida L., Petley E.V. (2020). Macrophage-Derived CXCL9 and CXCL10 Are Required for Antitumor Immune Responses Following Immune Checkpoint Blockade. Clin. Cancer Res..

[38] Reschke R., Yu J., Flood B.A., Higgs E.F., Hatogai K., Gajewski T.F. (2021). Immune Cell and Tumor Cell-Derived CXCL10 Is Indicative of Immunotherapy Response in Metastatic Melanoma. J. Immunother. Cancer.

[39] Aliberti J., Reis e Sousa C., Schito M., Hieny S., Wells T., Huffnagle G.B., Sher A. (2000). CCR5 Provides a Signal for Microbial Induced Production of IL-12 by CD8 Alpha+ Dendritic Cells. Nat. Immunol..

[40] Dorner B.G., Dorner M.B., Zhou X., Opitz C., Mora A., Güttler S., Hutloff A., Mages H.W., Ranke K., Schaefer M. (2009). Selective Expression of the Chemokine Receptor XCR1 on Cross-Presenting Dendritic Cells Determines Cooperation with CD8+ T Cells. Immunity.

[41] Fuertes M.B., Kacha A.K., Kline J., Woo S.-R., Kranz D.M., Murphy K.M., Gajewski T.F. (2011). Host Type I IFN Signals Are Required for Antitumor CD8+ T Cell Responses through CD8α+ Dendritic Cells. J. Exp. Med..

[42] Garofalo C., De Marco C., Cristiani C.M. (2021). K Cells in the Tumor Microenvironment as New Potential Players Mediating Chemotherapy Effects in Metastatic Melanoma. N. Front. Oncol..

[43] McKay K., Moore P.C., Smoller B.R., Hiatt K.M. (2011). Association between natural killer cells and regression in melanocytic lesions. Hum. Pathol..

[44] Reschke R., Dumann K., Ziemer M. (2022). Risk Stratification and Clinical Characteristics of Patients with Late Recurrence of Melanoma (>10 Years). J. Clin. Med..

[45] Mittal D., Vijayan D., Putz E.M., Aguilera A.R., Markey K.A., Straube J., Kazakoff S., Nutt S.L., Takeda K., Hill G.R. (2017). Interleukin-12 from CD103^+^ Batf3-Dependent Dendritic Cells Required for NK-Cell Suppression of Metastasis. Cancer Immunol. Res..

[46] Mattei F., Schiavoni G., Belardelli F., Tough D.F. (2001). IL-15 is expressed by dendritic cells in response to type I IFN, double-stranded RNA, or lipopolysaccharide and promotes dendritic cell activation. J. Immunol..

[47] Bergamaschi C., Pandit H., Nagy B.A., Stellas D., Jensen S.M., Bear J., Cam M., Valentin A., Fox B.A., Felber B.K. (2020). Heterodimeric IL-15 delays tumor growth and promotes intratumoral CTL and dendritic cell accumulation by a cytokine network involving XCL1, IFN-γ, CXCL9 and CXCL10. J. Immunother. Cancer.

[48] Reschke R., Gajewski T.F. (2022). CXCL9 and CXCL10 bring the heat to tumors. Sci. Immunol..

[49] Hoch T., Schulz D., Eling N., Gómez J.M., Levesque M.P., Bodenmiller B. (2022). Multiplexed imaging mass cytometry of the chemokine milieus in melanoma characterizes features of the response to immunotherapy. Sci. Immunol..

[50] Sánchez-Paulete A.R., Teijeira Á., Quetglas J.I., Rodríguez-Ruiz M.E., Sánchez-Arráez Á., Labiano S., Etxeberria I., Azpilikueta A., Bolaños E., Ballesteros-Briones M.C. (2018). Intratumoral Immunotherapy with XCL1 and SFlt3L Encoded in Recombinant Semliki Forest Virus–Derived Vectors Fosters Dendritic Cell–Mediated T-Cell Cross-Priming. Cancer Res..

[51] Williford J.-M., Ishihara J., Ishihara A., Mansurov A., Hosseinchi P., Marchell T.M., Potin L., Swartz M.A., Hubbell J.A. (2019). Recruitment of CD103^+^ Dendritic Cells via Tumor-Targeted Chemokine Delivery Enhances Efficacy of Checkpoint Inhibitor Immunotherapy. Sci. Adv..

[52] Antonicelli F., Lorin J., Kurdykowski S., Gangloff S.C., Le Naour R., Sallenave J.M., Hornebeck W., Grange F., Bernard P. (2011). CXCL10 Reduces Melanoma Proliferation and Invasiveness in Vitro and in Vivo: CXCL10 and Melanoma Progression. Br. J. Dermatol..

[53] Yin P., Gui L., Wang C., Yan J., Liu M., Ji L., Wang Y., Ma B., Gao W.-Q. (2020). Targeted Delivery of CXCL9 and OX40L by Mesenchymal Stem Cells Elicits Potent Antitumor Immunity. Mol. Ther..

[54] Cervera-Carrascon V., Quixabeira D.C.A., Santos J.M., Havunen R., Zafar S., Hemminki O., Heiniö C., Munaro E., Siurala M., Sorsa S. (2020). Tumor Microenvironment Remodeling by an Engineered Oncolytic Adenovirus Results in Improved Outcome from PD-L1 Inhibition. OncoImmunology.

[55] Eckert E.C., Nace R.A., Tonne J.M., Evgin L., Vile R.G., Russell S.J. (2019). Generation of a Tumor-Specific Chemokine Gradient Using Oncolytic Vesicular Stomatitis Virus Encoding CXCL9. Mol. Ther. Oncolytics.

[56] Liu Z., Ravindranathan R., Kalinski P., Guo Z.S., Bartlett D.L. (2017). Rational Combination of Oncolytic Vaccinia Virus and PD-L1 Blockade Works Synergistically to Enhance Therapeutic Efficacy. Nat. Commun..

[57] Mowat C., Mosley S.R., Namdar A., Schiller D., Baker K. (2021). Anti-Tumor Immunity in Mismatch Repair-Deficient Colorectal Cancers Requires Type I IFN–Driven CCL5 and CXCL10. J. Exp. Med..

[58] Kobayashi H., Nobeyama Y., Nakagawa H. (2015). Tumor-Suppressive Effects of Natural-Type Interferon-β through CXCL10 in Melanoma. Biochem. Biophys. Res. Commun..

[59] Peng W., Liu C., Xu C., Lou Y., Chen J., Yang Y., Yagita H., Overwijk W.W., Lizée G., Radvanyi L. (2012). PD-1 blockade enhances T cell migration to tumors by elevating IFN-γ inducible chemokines. Cancer Res..

[60] Orr B., Mahdi H., Fang Y., Strange M., Uygun I., Rana M., Zhang L., Mora A.S., Pusateri A., Elishaev E. (2022). Phase I trial combining chemokine-targeting with loco-regional chemoimmunotherapy for recurrent, platinum-sensitive ovarian cancer shows induction of CXCR3 ligands and markers of type 1 immunity. Clin. Cancer Res..

[61] Mansurov A., Hosseinchi P., Chang K., Lauterbach A.L., Gray L.T., Alpar A.T., Budina E., Slezak A.J., Kang S., Cao S. (2022). Masking the immunotoxicity of interleukin-12 by fusing it with a domain of its receptor via a tumour-protease-cleavable linker. Nat. Biomed. Eng..

[62] Antonelli A., Ferrari S.M., Corrado A., Ferrannini E., Fallahi P. (2014). CXCR3, CXCL10 and Type 1 Diabetes. Cytokine Growth Factor Rev..

[63] Boniface K., Jacquemin C., Darrigade A.-S., Dessarthe B., Martins C., Boukhedouni N., Vernisse C., Grasseau A., Thiolat D., Rambert J. (2018). Vitiligo Skin Is Imprinted with Resident Memory CD8 T Cells Expressing CXCR3. J. Investig. Dermatol..

[64] Han J.H., Suh C.-H., Jung J.-Y., Ahn M.-H., Han M.H., Kwon J.E., Yim H., Kim H.-A. (2017). Elevated Circulating Levels of the Interferon-γ-Induced Chemokines Are Associated with Disease Activity and Cutaneous Manifestations in Adult-Onset Still’s Disease. Sci. Rep..

[65] Ito T. (2013). Recent Advances in the Pathogenesis of Autoimmune Hair Loss Disease Alopecia Areata. Clin. Dev. Immunol..

[66] Loos T., Dekeyzer L., Struyf S., Schutyser E., Gijsbers K., Gouwy M., Fraeyman A., Put W., Ronsse I., Grillet B. (2006). TLR Ligands and Cytokines Induce CXCR3 Ligands in Endothelial Cells: Enhanced CXCL9 in Autoimmune Arthritis. Lab. Investig..

[67] Luoma A.M., Suo S., Williams H.L., Sharova T., Sullivan K., Manos M., Bowling P., Hodi F.S., Rahma O., Sullivan R.J. (2020). Molecular Pathways of Colon Inflammation Induced by Cancer Immunotherapy. Cell.

[68] Reschke R., Gussek P., Boldt A., Sack U., Köhl U., Lordick F., Gora T., Kreuz M., Reiche K., Simon J.C. (2021). Distinct Immune Signatures Indicative of Treatment Response and Immune-Related Adverse Events in Melanoma Patients under Immune Checkpoint Inhibitor Therapy. Int. J. Mol. Sci..

[69] Amatschek S., Lucas R., Eger A., Pflueger M., Hundsberger H., Knoll C., Grosse-Kracht S., Schuett W., Koszik F., Maurer D. (2011). CXCL9 Induces Chemotaxis, Chemorepulsion and Endothelial Barrier Disruption through CXCR3-Mediated Activation of Melanoma Cells. Br. J. Cancer.

[70] Hong W.X., Sagiv-Barfi I., Czerwinski D.K., Sallets A., Levy R. (2022). Neoadjuvant Intratumoral Immunotherapy with TLR9 Activation and Anti-OX40 Antibody Eradicates Metastatic Cancer. Cancer Res..

[71] Mirzaei H., Salehi H., Oskuee R.K., Mohammadpour A., Mirzaei H.R., Sharifi M.R., Salarinia R., Darani H.Y., Mokhtari M., Masoudifar A. (2018). The Therapeutic Potential of Human Adipose-Derived Mesenchymal Stem Cells Producing CXCL10 in a Mouse Melanoma Lung Metastasis Model. Cancer Lett..

[72] Xia M., Chen J., Meng G., Shen H., Dong J. (2021). CXCL10 Encoding SynNotch T Cells Enhance Anti-Tumor Immune Responses without Systemic Side Effect. Biochem. Biophys. Res. Commun..

[73] Ma Y., Liu Y., Zhi Y., Wang H., Yang M., Niu J., Zhao L., Wang P. (2022). Delivery of CXCL9/10/11 Plasmid DNAs Promotes the Tumor-Infiltration of T Cells and Synergizes with PD1 Antibody for Treating Lung Cancer. Cancer Nanotechnol..

[74] Reschke R., Jäger I., Mehnert-Theuerkauf A., Ziemer M. (2021). Therapy understanding and health related quality of life in stage III/IV melanoma patients treated with novel adjuvant therapies. J. Dtsch. Dermatol. Ges..

