# Correction: Reschke, R.; Olson, D.J. Leveraging STING, Batf3 Dendritic Cells, CXCR3 Ligands, and Other Components Related to Innate Immunity to Induce a “Hot” Tumor Microenvironment That Is Responsive to Immunotherapy. *Cancers* 2022, *14*, 2458

**DOI:** 10.3390/cancers16061234

**Published:** 2024-03-21

**Authors:** Robin Reschke, Daniel J. Olson

**Affiliations:** 1 Department of Pathology, University of Chicago, Chicago, IL 60637, USA; 2 Department of Medicine, University of Chicago, Comprehensive Cancer Center, Chicago, IL 60637, USA

The authors would like to make the following corrections to their published paper [1]:

1. Change the last two sentences of Simple Summary into one sentence: “In particular, Batf3-lineage DCs are highly efficient in priming and recruiting effector T cells”.

2. Shorten the third last sentence of the abstract to the following: “CD103^+^ DCs are integral for priming and recruiting of effector T cells”.

3. Revise the heading of Section 3 from “Chemokines in The Tumor Microenvironment” to “Batf3 DCs Initiating a Chemokine-Cytokine Network in the TME”. Add the following sentences to the end of the second paragraph in Section 3:

“NK cells themselves can also be recruited via CXCL9-11 because they express CXCR3 on their surface [42]. In the TME, they are involved in killing cancer stem cells and thereby prevent early and late metastases [43,44]. NK cells additionally secrete IFNγ and TNFα [42]. For optimal tumor control, it has been found that IL12 exerted from Batf3 DCs is the premise for effective NK cell function and production of IFNγ [45]. Only Batf3 DCs were able to produce sufficient IL12 and achieve NK cell-mediated control of metastasis. Next to IL12, IL15 represents another important cytokine that can be produced by dendritic cells upon activation with type I IFN [46]. IL15 in turn activates NK cells and cytotoxic T cells and increases the production of IFNγ. Additionally, it boosts the production of CXCL9-11 in Batf3 DCs, most likely indirectly through increased IFNγ production [47]. Interestingly, a very recent study found that “exhausted” T cells in the tumor microenvironment that were actively recruited via CXCL9 and CXCL10 can also be considered chemokine-producing cells. They express checkpoint molecules such as LAG3 but also secrete CCL4 and CXCL13 and thereby attract more Batf3 DCs and B cells into the tumor microenvironment [48,49]. Accumulated B cells can form tertiary lymphoid structures and contribute to anti-tumor immunity. This complex chemokine/cytokine network starting with Batf3 DCs could potentially be targeted at various points with personalized medicine approaches, many of which are already being tested in clinical studies”.

4. Adjust Figure 3 as seen below:

5. Change the title of Section 3.1 from “Inducing Chemokines” to “Inducing Chemokines and Cytokines”, and add the following content to the original text, starting from the end of the paragraph:

…“A combination treatment consisting of chemotherapy (cisplatin), COX-2 inhibitor (celecoxib), type I IFN, and a TLR3 agonist (rintalomid) also led to successful induction of CXCL9-11 in patients with epithelial ovarian cancer [60]. In the future, this approach will be combined with DC vaccination to achieve optimal tumor control of “cold” tumors via cytotoxic T cell recruitment. In mice, the administration of heterodimeric IL-15 resulted in increased levels of XCL1, IFNγ, and CD103+ DCs, as well as CXCL9/10 production, and the recruitment of NK and T cells [47]. Another attractive avenue would be to leverage radiation therapy to induce cytokine/chemokine production. Radiation therapy in melanoma-bearing mice induced Type I IFN and CXCL10 production by myeloid cells because cytosolic dsDNA released from dying cancer cells activates the STING pathway in dendritic cells [61].

All these approaches constitute important new treatment avenues for “cold” tumors. However, these strategies of chemokine and cytokine induction only work well when key innate immune cells such as Batf3 DCs are present in the TME. Another limiting factor is the right formulation and delivery of these components. Systemic application or an increased dosage of CXCL9-11, for example, may lead to unwanted immune-related adverse events. Severe adverse events can lead to the discontinuation of checkpoint blockade therapy. Innovative delivery strategies might help circumvent toxicity. In a mouse model, masking IL12 by fusing it to a domain of the IL12 receptor prevented systemic toxicity despite intravenous application [62]. With this modification, the anti-tumor effect remained intact”.

6. Revise the title of Section 3.2 from “Adverse Effects Caused by Chemokines” to “Adverse Effects Caused by Chemokines and Cytokines”. Revise the first sentence of Section 3.2 from “Systemic upregulation of CXCL9/10/11 can contribute to inflammation and autoimmunity such as Alopecia areata, Vitiligo, autoimmune arthritis, type 1 diabetes, or adult-onset Still’s disease” to “Systemic upregulation of CXCL9/10/11 can contribute to inflammation and autoimmunity such as Alopecia areata, Vitiligo, autoimmune arthritis, type 1 diabetes, or adult-onset Still’s disease as well as immunotherapy-induced toxicity [62–66]”. Add the sentence “Increased levels of CXCL9-11 in the blood during checkpoint blockade therapy were also associated with occurrence of irAE [68]” as the third sentence in Section 3.2. Add “Similarly, systemic administration of cytokines can lead to severe and life-threatening toxicity. IL12, for example, results in an extensive production of systemic IFN-γ from NK cells [62]. Accordingly, potential off-tumor effects of systemic administration of cytokines must always be considered in clinical trial designs of these therapies” as the last sentence in Section 3.2.

7. Revise Table 1 title and content to the following version (added lines TLR3, IL-12 (mouse) and IL-15 (mouse); removed lines TLR3+, Flt3L+, B7-H3+, 4-1BBL+, and IL-12 (human)). Add it’s citation after the sentence “Two recent reports propose alternate administration routes for the more stable STING agonists named MSA-2 and SR-717 configured in a closed confirmation [28,29]” in Section 2.

8. Delete contents of the original Section 4 and Figure 4.

9. Add “Checkpoint blockade therapy is already used for many metastasized solid tumors but is also well accepted in the adjuvant setting for, e.g., melanoma patients [74]” as the last sentence in Section 4, “Conclusions”.

10. Delete the original references 59–76, 81, and 82; add some new ones as references 42–49, 60, 61, 68 and 74; the original reference numbers starting from new reference 42 are also updated. The other references are also updated accordingly.

- 42. Garofalo, C.; De Marco, C.; Cristiani, C.M. NK Cells in the Tumor Microenvironment as New Potential Players Mediating Chemotherapy Effects in Metastatic Melanoma. *Front. Oncol.* **2021**, *12*, 754541. https://doi.org/10.3389/fonc.2021.754541.
- 43. McKay, K.; Moore, P.C.; Smoller, B.R.; Hiatt, K.M. Association between natural killer cells and regression in melanocytic lesions. *Hum. Pathol.* **2011**, *42*, 1960–1964. https://doi.org/10.1016/j.humpath.2011.02.019.
- 44. Reschke, R.; Dumann, K.; Ziemer, M. Risk Stratification and Clinical Characteristics of Patients with Late Recurrence of Melanoma (>10 Years). *J. Clin. Med.* **2022**, *11*, 2026. https://doi.org/10.3390/jcm11072026.
- 45. Mittal, D.; Vijayan, D.; Putz, E.M.; Aguilera, A.R.; Markey, K.A.; Straube, J.; Kazakoff, S.; Nutt, S.L.; Takeda, K.; Hill, G.R.; et al. Interleukin-12 from CD103+ Batf3-Dependent Dendritic Cells Required for NK-Cell Suppression of Metastasis. *Cancer Immunol. Res.* **2017**, *5*, 1098–1108. https://doi.org/10.1158/2326-6066.
- 46. Mattei, F.; Schiavoni, G.; Belardelli, F.; Tough, D.F. IL-15 is expressed by dendritic cells in response to type I IFN, double-stranded RNA, or lipopolysaccharide and promotes dendritic cell activation. *J. Immunol.* **2001**, *167*, 1179–1187. https://doi.org/ 10.4049/jimmunol.167.3.1179.
- 47. Bergamaschi, C.; Pandit, H.; Nagy, B.A.; Stellas, D.; Jensen, S.M.; Bear, J.; Cam, M.; Valentin, A.; Fox, B.A.; Felber, B.K.; et al. Heterodimeric IL-15 delays tumor growth and promotes intratumoral CTL and dendritic cell accumulation by a cytokine network involving XCL1, IFN--γ, CXCL9 and CXCL10. *J. Immunother. Cancer* **2020**, *8*, e000599. https://doi.org/10.1136/jitc-2020-000599.
- 48. Reschke, R.; Gajewski, TF. CXCL9 and CXCL10 bring the heat to tumors. *Sci. Immunol.* **2022**, *7*, eabq6509. https://doi.org/10.1126/sciimmunol.abq6509.
- 49. Hoch, T.; Schulz, D.; Eling, N.; Gómez, J.M.; Levesque, M.P.; Bodenmiller, B. Multiplexed imaging mass cytometry of the chemokine milieus in melanoma characterizes features of the response to immunotherapy. *Sci. Immunol.* **2022**, *7*, eabk1692. https://doi.org/10.1126/sciimmunol.abk1692.
- 60. Orr, B.; Mahdi, H.; Fang, Y.; Strange, M.; Uygun, I.; Rana, M.; Zhang, L.; Mora, A.S.; Pusateri, A.; Elishaev, E.; et al. Phase I trial combining chemokine-targeting with loco-regional chemoimmunotherapy for recurrent, platinum-sensitive ovarian cancer shows induction of CXCR3 ligands and markers of type 1 immunity. *Clin. Cancer Res.* **2022**, *28*, 2038–2049.
- 61. Mansurov, A.; Hosseinchi, P.; Chang, K.; Lauterbach, A.L.; Gray, L.T.; Alpar, A.T.; Budina, E.; Slezak, A.J.; Kang, S.; Cao, S.; et al. Masking the immunotoxicity of interleukin-12 by fusing it with a domain of its receptor via a tumour-protease-cleavable linker. *Nat. Biomed. Eng.* **2022**, *6*, 819–829. https://doi.org/10.1038/s41551-022-00888-0.
- 68. Reschke, R.; Gussek, P.; Boldt, A.; Sack, U.; Köhl, U.; Lordick, F.; Gora, T.; Kreuz, M.; Reiche, K.; Simon, J.C.; et al. Distinct Immune Signatures Indicative of Treatment Response and Immune-Related Adverse Events in Melanoma Patients under Immune Checkpoint Inhibitor Therapy. *Int. J. Mol. Sci.* **2021**, *22*, 8017. https://doi.org/10.3390/ijms22158017.
- 74. Reschke, R.; Jäger, I.; Mehnert-Theuerkauf, A.; Ziemer, M. Therapy understanding and health related quality of life in stage III/IV melanoma patients treated with novel adjuvant therapies. *J. Dtsch. Dermatol. Ges.* **2021**, *19*, 215–221. https://doi.org/10.1111/ddg.14317.

The authors apologize for any inconvenience caused and state that the results and scientific conclusions are unaffected. This correction was peer reviewed by the previous referees and approved by the Academic Editor. The original publication has also been updated.

## Figures and Tables

**Figure 3:**
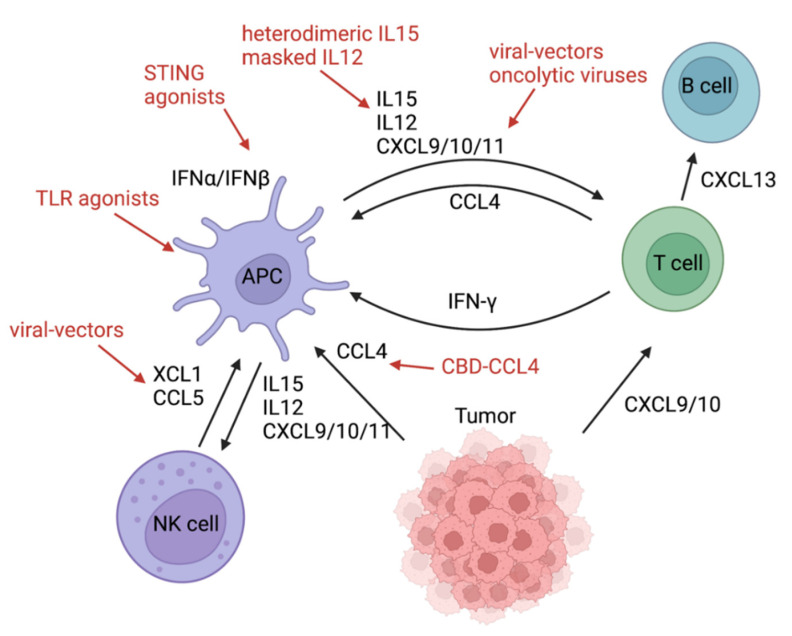
Chemokine/cytokine network in the tumor microenvironment. Tumor-derived CCL4 can attract Batf3 DCs, and CXCL9/10 can recruit cytotoxic T cells to the tumor microenvironment. Natural Killer Cells attract Batf3 DCs via XCL1 and CCL5. CCL4 can be administered with a fusion protein consisting of CCL4 and the collagen-binding domain (CBD) of von Willebrand factor and XCL1 with a viral vector. APCs (in particular Batf3 DCs) can produce CXCL9/10/11, IL12, and IL15 and can recruit and activate NK and cytotoxic T cells. In turn, IFN-γ produced by CTLs can stimulate APCs. APCs are activated by STING or TLR agonists, which can contribute to CXCL9/10/11 production via IFNα/β. CXCL9/10/11 can be induced by oncolytic viruses or delivered with the help of virus-based vectors.

**Table 1:** Induction and delivery of components related to the innate immune system that synergize with immunotherapy: summary of recent preclinical and clinical studies.

Components	Function	Species	Delivery Route/Therapeutic Agent	Year/Citation
**STING**	Sensing tumor-derived DNA, DC maturation, and CTL priming	mouse	systemic administration of SR-717 in a “closed” conformation	2020 [28]
		mouse	oral administration of MSA-2 in a “closed” conformation	2020 [29]
		mouse	engineered extracellular vesicle exogenously loaded with cyclic dinucleotide	2021 [30]
		human	intravenous infusion of TAK-676	ongoing NCT04420884
		human	intravenous infusion of SB 11285	ongoing NCT04096638
		human	intravenous infusion of SNX281	ongoing NCT04609579
		human	intratumoral injection of CDK-002	ongoing NCT04592484
**TLR9**	sensing tumor-derived DNA, DC maturation, and CTL priming	human	intratumoral injection of Vidutolimod	2021 [24]
		mouse	intratumoral injection CpG oligodeoxynucleotide (TLR9 ligand) and an antibody against OX40	2022 [70]
**TLR3**	maturation and CTL priming	human	intraperitoneal injection of rintalomid (TLR3 agonist), celecoxib, and cisplatin	2022 [60]
**VEGF**	local lymphangiogenesis, immune cell trafficking, and CTL activation	mouse	injection of “VEGFC vax”	2021 [35]
**XCL1+** **Flt3L**	DC recruitment and expansion	mouse	intratumoral injection of XCL1 and SFlt3L encoded in recombinant Semliki Forest virus-derived vectors	2018 [50]
**CCL4**	DC recruitment	mouse	intravenous administration of a fusion protein of CCL4 and the collagen-binding domain of von Willebrand factor	2019 [51]
**CXCL9/10/11**	CTL recruitment	mouse	CXCL9 and OX40L	2020 [53]
		mouse	intravenous injection of oncolytic vesicular stomatitis virus encodes CXCL9	2020 [55]
		mouse	genetically engineered mesenchymal stem cells producing CXCL10	2018 [71]
		humanized mouse	injection of CXCL10-producing SynNotch T cells	2021 [72]
		mouse	intravenous delivery of CXCL9/10/11 plasmids by nanoparticles	2022 [73]
		human	NG-641 is an oncolytic adenoviral vector which expresses a FAP-TAc antibody together with an immune enhancer module (CXCL9/CXCL10/IFNα).	ongoing NCT04053283
**IL12**	DC activation/IFNγ production of T and NK cells	mouse	intravenous injection of IL12 fused to a domain of the IL12 receptor	2022 [61]
**IL15**	DC recruitment/activation and downstream recruitment of CTLs/NK cells	mouse	intraperitoneal injection of heterodimeric IL-15	2020 [47]
